# *Pydidas*: a tool for automated X-ray diffraction data analysis

**DOI:** 10.1107/S160057672500398X

**Published:** 2025-06-16

**Authors:** Malte Storm, Peter Staron, Christina Krywka

**Affiliations:** a Institute of Materials Physics, Helmholtz-Zentrum Hereon GmbH, Geesthacht, Germany; DESY, Hamburg, Germany

**Keywords:** X-ray diffraction, XRD analysis, software

## Abstract

The processing and analysis of X-ray diffraction (XRD) data at synchrotrons is often left to the user groups, which limits the user base to groups with a background in analyzing XRD data. *Pydidas* is a new Python package for processing XRD data. It provides an easy and intuitive interface and versatile processing options with the aim of being accessible to non-experts in XRD analysis. A graphical user interface (GUI) allows users to perform the full pipeline of data browsing, experiment calibration, workflow setup, processing and visualization in a single tool. In addition, *pydidas*’ logic is decoupled from the GUI and it can be fully used from within scripts or embedded into other processing pipelines. The *pydidas* processing pipeline is assembled from individual plugins which perform specific processing steps. This modular design allows for very versatile pipelines covering a wide range of applications. To improve the usability even further, custom plugins can be integrated in the *pydidas* workflow to allow specialized processing steps.

## Abstract 2

*Pydidas* is a new Python package for processing X-ray diffraction data, offering a user-friendly interface and versatile processing options. It includes a graphical user interface for the entire data processing pipeline and is intended to be easily accessible for non-experts.

## 1. Introduction

*Pydidas* is software developed by Helmholtz-Zentrum Hereon to improve data processing at its X-ray diffraction (XRD) oriented beamlines operated at the PETRA III synchrotron radiation source (DESY, Hamburg, Germany). Hereon operates multiple beamlines, among which are the P03 nanofocus endstation for nano-diffraction experiments and the P07 high-energy materials science beamline (HEMS), which are dedicated to diffraction experiments. At both beamlines, *in situ* or *operando* sample environments are frequently used. Especially fast *in situ* or *operando* experiments require fast feedback (in the form of data processing) to optimize the beamtime usage during experiments. In addition, the increased data rates of all experiments require novel solutions to allow more user-friendly and automated data processing by beamline users.

Historically, data reduction and pre-processing of XRD datasets has been left in the hands of user groups which limited the user community to those groups who had the capabilities and personnel to perform diffraction data analysis. To make XRD methods more accessible for user groups with little to no experience in XRD data analysis and to attract more users from industry, suitable data analysis tools must be made available. So far, this objective has been successfully implemented only in selected areas, reflected by the fact that different techniques at synchrotron beamlines offer hugely different user experiences. Specific techniques like macromolecular crystallography already offer highly automated solutions for both experiments and analysis which allow users without any prior technical knowledge to perform successful experiments (Gabadinho *et al.*, 2010 ▸; *ISPyB*, https://github.com/ispyb/ISPyB).

A wide range of software tools exist for XRD data analysis, but these are usually either designed for expert users or tailored to specific use cases. Python packages for efficient (azimuthal) integration like *pyFAI* (Kieffer & Karkoulis, 2013 ▸; Kieffer & Wright, 2013 ▸; https://github.com/silx-kit/pyFAI) or *azint* (Jensen *et al.*, 2022 ▸; https://github.com/maxiv-science/azint) allow expert users a high degree of flexibility and scripting options as well as very performant computational implementations. While *pyFAI*, for example, also offers graphical user interfaces (GUIs), these are limited to *pyFAI*’s core role of calibration and integration.

In addition, a wide range of tools have been developed which aim to integrate GUIs and processing functionality. Usually these include additional tools like data browsing or advanced fitting routines. *FIT2D* (Hammersley *et al.*, 1996 ▸; Hammersley, 2016 ▸), created by the ESRF, was one of the first generic tools widely available for a large user community and it is a testament to the software that it is still used today even though it is no longer actively supported. A variety of other analysis software tools have been developed over the years, either driven by facilities or by specific techniques and their requirements. Facility-specific tools include *DAWN*, created by Diamond Light Source (Filik *et al.*, 2017 ▸), *Xi-Cam* (https://github.com/Xi-CAM/Xi-cam), developed by the Advanced Light Source, and *GSAS-II* (Toby & Von Dreele, 2013 ▸), created by the Advanced Photon Source. Other tools have been developed with a particular expert community in mind, like *Dioptas* (Prescher & Prakapenka, 2015 ▸) for high-pressure powder diffraction or *DPDAK* (Benecke *et al.*, 2014 ▸) for small-angle X-ray scattering and grazing-incidence small-angle X-ray scattering. Except for *FIT2D* and *DAWN*, which both have their own integration algorithms, all these tools use the *pyFAI* engine.

Most of these tools offer GUIs which are, however, designed primarily for expert users familiar with diffraction techniques and the required analysis steps. In particular, the wealth of possible parameters required to set up the processing can be overwhelming for inexperienced users.

To improve the analysis throughput of both our expert and first-time beamline users, we need software with an intuitive and user-friendly interface. It should also support fast, parallel processing of large datasets, potentially containing tens of thousands of images, due to the continuously increasing data rates at beamlines. Existing software often falls short in one or more of these areas. *Pydidas* was developed to address this gap by offering software designed to be intuitive and accessible to users with little or no experience in XRD data analysis, while still providing expert users with low-level access to all essential parameters.

## 2. Preparation of an XRD analysis in *pydidas*

The configuration for workflow processing has been split into the three individual categories of *experiment*, *scan* and *workflow*. The parameters in these categories correspond to answers to the questions of ‘How did you measure your data?’ for the *experiment*, ‘What did you measure?’ for the *scan* and ‘How do you want to process your data?’ for the *workflow*.

This division allows the configuration to be reused extensively for later or repeated processing, for example of a scan series during an experiment, or reuse of the same workflow during other beamtimes.

Covering a wide range of different applications and use cases requires a high flexibility with respect to the design of the analysis workflow. Therefore, the *pydidas* processing pipeline is based on a sequence of individual plugins which each only handle a specific processing step. The full pipeline is then assembled by combining individual plugins to the full processing workflow.

Table 1 ▸ gives an overview of the items in the three categories. Except for specific filenames, for example, subtracting a background image, no filenames are defined in the workflow definition. The global detector mask file, if applicable, is defined in the experiment definition while the raw data path and the filenaming pattern are part of the scan definition. Batch processing of different scans only requires updating the paths in the scan configuration which facilitates the processing setup for inexperienced users. Also, this makes reusing workflows between experiments or facilities straightforward as the filenames and paths are all defined in the scan section.

These global configurations are considered to be static during an experiment and analysis. This assumption allows more efficient handling of these, for example by loading detector masks once at the start. However, the architecture allows the use of local instances instead of the global configuration. For example, plugins can use a dynamic detector mask instead of the global one.

Because *pydidas* uses *pyFAI* for data reduction (most notably the integration of 2D datasets), it follows the *pyFAI* definitions for the detector and PONI geometry (point of normal incidence), which describes the detector by the point where the beam from the sample would hit the detector at a right angle (Kieffer & Wright, 2013 ▸).

## 3. Design considerations

### 3.1. General design

*Pydidas* is a Python package which uses *Qt* (https://www.qt.io) through the *qtpy* bindings (https://github.com/spyder-ide/qtpy) and is compatible with *Qt* versions 5 and 6. The default implementation is currently *PyQt5* and *pydidas* has also been tested using *PySide6*. *Qt* is used both for the GUI and, in some circumstances, for internal communication using *Qt*’s signals and slots. For example, signals are used when running workflows with parallel processing for triggering internal actions (*e.g.* saving to disk).

A well designed GUI is a key requirement to make *pydidas* easily accessible to inexperienced users with no prior experience with command-line interfaces. Also, data visualization and interactive tasks require a graphical interface. Therefore, *pydidas* was primarily designed around the graphical interface. Nevertheless, care has been taken to separate the core functionality from the GUI and to allow *pydidas* to be used from the command line or scripts. Only interactive functionality which requires graphical feedback is available exclusively from the GUI.

### 3.2. User experience

The explicit aspiration of *pydidas* is to allow full processing workflows of diffraction data within a single software tool. Therefore, users only have to use two programs: the beamline control software for experimental control and *pydidas* for data analysis.

**Figure 1:**
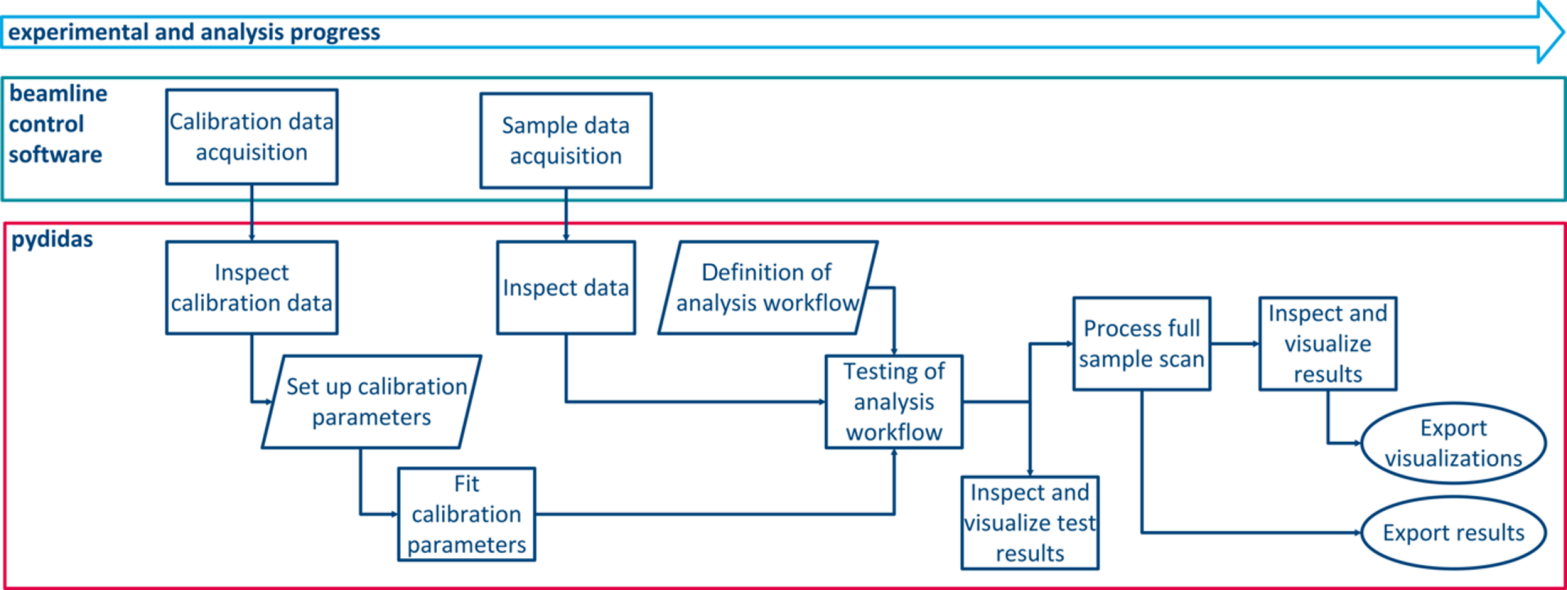
Flow-chart diagram of all steps required to perform a typical analysis of XRD data. The main idea is to provide all required functionality within only two interfaces, the beamline control software for data acquisition and *pydidas* (shown in the red box) for data analysis. The two boxes for definition and testing of analysis workflows are highlighted in blue and are shown in more detail in Fig. 2 ▸.

**Figure 2:**
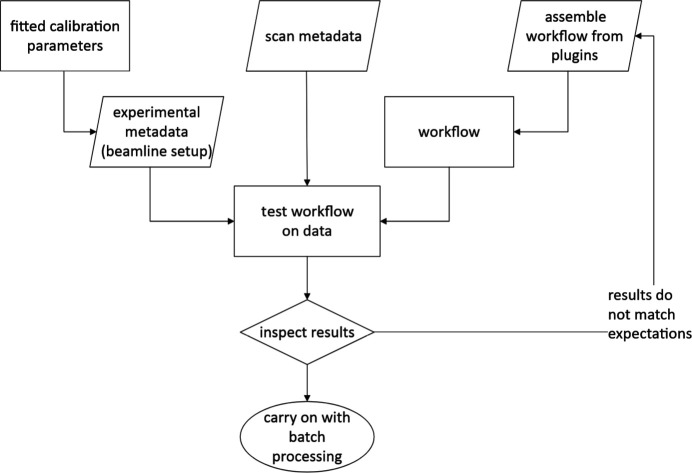
Detailed flow-chart diagram of the XRD analysis configuration which corresponds to the ‘definition of analysis workflow’ and ‘testing of analysis workflow’ steps from Fig. 1 ▸. The experimental setup, scan metadata and workflow are individually defined by the user. Together, they make up the configuration required for XRD data analysis.

A minimal workflow for any data analysis consists of data exploration, experiment calibration, definition of the analysis workflow and visualization of the results, as shown schematically in Fig. 1 ▸. All these steps are available via the *pydidas* GUI. The detailed process for setting up the analysis configuration is depicted in Fig. 2 ▸.

Global configurations of *pydidas* are stored on a per-user basis using the functionality available in *Qt* which allows for a convenient and platform-independent solution. The configuration of user preferences like default colormaps, font or font size improves the user experience and also allows different visual requirements to be catered for.

In addition, *pydidas* allows storage of the state of the GUI and configuration manually at any time in files and automatically when closing the program. This allows users to interrupt complex analysis processes and conveniently resume working after restarting the GUI.

An important part of the user experience is also knowing how to obtain support, if required. The first step should always be the documentation. The documentation for *pydidas* is available as webpages (https://hereon-gems.github.io/pydidas) and it includes content for user groups from novice users to experts who want to develop and integrate their custom plugins. In addition, a local version of the documentation is included with the Python package. This allows both offline access (*e.g.* during travel or in an isolated environment) as well as direct integration with the GUI. Asking for help in the GUI (by pressing F1) will open the local documentation for the current view.

### 3.3. Supported data formats

HDF5 is the *de facto* standard file format for modern area detectors at synchrotrons as it allows both native data compression and storage of multiple detector images in a single file which eases the burden on the file system. However, many detectors still write other data formats (*e.g.* TIFF or binary) or – in the case of point or line detectors – even in ASCII formats. *Pydidas* is designed to support most common data formats (HDF5, TIFF, binary, formats supported by ESRF’s FabIO) and the modular structure of workflows allows users to easily implement loaders for additional data formats.

The flexibility of the HDF5 container allows metadata to be attached to all result files written by *pydidas*, thus improving clarity and reproducibility. *Pydidas* uses HDF5 as its native format for results and written results adhere to the NeXus definition (Könnecke *et al.*, 2015 ▸; https://www.nexusformat.org/) for data annotations. Reproducibility is improved by including metadata about the configuration in the results, which allows users to repeat the exact processing, assuming access to the raw data. Nevertheless, some other software tools (*e.g.* for texture or Rietveld analysis) require data in other formats, for example ASCII. We acknowledge the need to preserve compatibility with other tools and *pydidas* includes a plugin for ASCII export. Exporters for other data formats can also be added through custom plugins.

### 3.4. Utilities

Next to processing workflows, additional utilities are required at many points during data analysis. For example, calibration data or background images might need to be averaged. Data might have to be modified with mathematical and logical operations, *e.g.* by multiplication and subtraction to allow for background subtraction which accounts for varying sample absorption. Detector masks might need to be created or modified to exclude damaged pixels or inter-module gaps from data analysis. All these tasks are required to prepare the data evaluation. Hence, these tools have also been integrated into the *pydidas* GUI.

### 3.5. Flexibility

Another key design consideration is flexibility. The variety of possible data acquisition and analysis schemes requires very broad analysis capabilities. While *pydidas* tries to cover a large portion of standard use cases, we acknowledge that covering all possible cases is impossible. Consequently, *pydidas* allows users to create their own plugins for extending the analysis capabilities and make them available within *pydidas*. In this way, new file formats can also be added without needing to modify *pydidas*’ source code. An example of a minimal plugin is given in Appendix *A*[App appa].

The GUI is designed in a modular fashion and even extending the GUI and adding new widgets is possible for users with some programming experience.

## 4. Software architecture

This section will detail the architectural design decisions which have been made to obtain a system which both is performant and complies with the design considerations discussed above.

### 4.1. Parameter handling

All configuration parameters (this includes processing parameters and plugin configurations) are managed through class objects, which allows parameters to be shared between programmatic objects and implementation of type-checking of inputs directly as they are entered. User input is thus automatically checked for wrong data types with custom exception handling to improve the user experience.

### 4.2. Processing workflows

Processing workflows are user-defined sequences composed of individual plugins that perform specific operations on the data. Splitting the processing into many small items gives a high flexibility in editing workflows. It also limits the necessary parameters for each plugin because most optional functionality can be moved to specific plugins. The processing workflow always starts with a single plugin which loads the data. Each plugin can pass its results to multiple plugins further down the processing workflow, called its children. This behavior allows branching workflows.

The plugin organization, calling and data transport between plugins is managed through a single object, which has been named ProcessingTree. This object allows for convenient access and management of plugins.

For *pydidas*, we have made the design decision to limit workflows to a strictly linear data flow. This decision was the result of an internal analysis of possible use cases and differs from other popular workflow engines like Orange (https://github.com/biolab/orange3) which is also based on *Qt* and comes with a GUI for the creation and customization of workflows. While the decision to use linear data workflows is a limitation in the sense that it prevents, for example, the implementation of feedback loops, it makes up for this by having a very simple interface for plugins which is in line with the demand for *pydidas* to be easily accessible on all scales. Also, having data flow only from one plugin to the next greatly reduces the risk of users accidentally misconfiguring the workflow by connecting incompatible plugins.

### 4.3. Export of results

Next to the final result of an analysis process (*e.g.* residual stress map), multiple intermediate results are typically generated along the way, all of which may carry important information to *e.g.* verify the plausibility of the final result. Therefore, processing results from all plugins and for all scan points are stored during runtime in one global object called WorkflowResults. By default, only plugins which have no children export their results, but data export can be enabled for all plugins, if required. Results can be accessed internally or exported to file. *Pydidas* exports results for each plugin in HDF5 format with the full processing metadata of experiment, scan and workflow as well as information about the *pydidas* version written in each resulting file. Therefore, each HDF5 result file alone includes all the information required to reproduce the full processing. While other formats for global result exports are not natively included, the *pydidas* architecture allows the easy addition of other output formats, if these are required by specific user communities. Additionally, output plugins can be used to export results from each scan point in the workflow. For example, *pydidas* includes a plugin to export results from each scan point to individual ASCII files.

### 4.4. Parallelization

To make efficient use of system resources, *pydidas* allows parallelization with multiple processes. While some packages, for example *pyFAI*, make use of CPU resources quite efficiently, file reading and other processing steps (*e.g.* fitting) are not very well parallelized and the full workflow benefits from processing multiple images at once. This argument is even stronger when using GPUs for *pyFAI*’s integrations and thereby freeing up CPU resources to be used for other tasks. When using GPUs for processing, the overhead for data transfer to the GPU is usually also significant compared with the processing time on the GPU, which leads to processing speed improvements when accessing the GPU in parallel from multiple processes.

*Pydidas* uses multiple, independent processes for batch processing scans. Communication between the controller and worker processes is handled through pipes. To allow efficient data transfer between processes, a shared memory array is used. Only indices to find data in the shared array need to be transferred, significantly increasing the communication speed between processes.

The *pydidas* parallelization is not based on other, existing parallelization engines like *Dask* (https://dask.org) but custom built. The main reason for a customized parallelization is the handling of results. Results are made available in the GUI as soon as they are available, which allows users to inspect results as they are computed and which is a requirement when visualizing, for example, *in situ* measurement results as the experiment progresses. Headless processing, for example by submitting to high-performance computing scheduling systems, has so far not been a development priority. Nevertheless, the *pydidas* architecture clearly separates the processing logic from the parallelization logic which allows *pydidas* processing workflows to also be run in other workflow engines, *e.g.Dask*, and use their parallelization routines.

### 4.5. GUI

The GUI is composed of individual widgets, called frames in *pydidas*. The GUI itself is an empty window and we have included a register_frame method. This gives an easy way to either create GUI instances with limited functionality or add custom frames.

The arrangement of these frames reflects the sequence of actions required to perform a typical data analysis. The main GUI includes classical menus but the main navigation is handled through toolbars on the left. A screenshot of the GUI after startup is given in Fig. S1 in the supporting information. Each frame includes a corresponding button in the taskbar for displaying it. To keep startup times performant and to allow for easy extensions, frames are not actually initialized until needed by the user. A list of the standard frames and a short description is given in Table 2 ▸.

### 4.6. Command-line interface

*Pydidas* is a Python package and, as such, all functionalities can be imported and used in scripts or interactive Python shells. Those *pydidas* objects that are required for user interactions (*e.g.* for processing setup) are designed to have identical interfaces to allow an easy start also from the command-line interface. Except for interactive elements, *pydidas* can be fully used from the command line. Parameters in all objects are accessible through similar methods and an example is given in Appendix *B*[App appb]. The *pydidas* documentation also describes the most used objects in detail.

## 5. Use case example: 2D strain scanning

This section is meant to showcase an analysis workflow in the *pydidas* GUI. It is based on data from Zeilinger *et al.* (2016 ▸) which they kindly shared. Following a general description of the experiment, the specific processing steps within *pydidas* are described. However, to not overburden this section with a large number of figures, the corresponding GUI screenshots are provided in the supporting information and only referenced herein. The data were acquired during a nano-indentation experiment at the Nanofocus Endstation of beamline P03 (PETRA III, DESY, Hamburg, Germany) (Krywka *et al.*, 2012 ▸). The sample was a nanocrystalline TiN hard coating deposited on a steel substrate using plasma-assisted chemical vapor deposition and nitriding. The 9 µm-thick TiN film consisted of nanocrystallites arranged in a columnar grain morphology. The sample was prepared by cutting a cross-sectional lamella consisting of the substrate and the film with a thickness of 40 µm in the beam direction and was analyzed in transmission via wide-angle X-ray diffraction. During data collection, the film was subjected to a microscopically localized mechanical load through a sharp diamond tip pushing into the surface. An X-ray beam focused to a size of 350 × 350 nm was used to record a grid of spatially resolved XRD datasets (referred to as a 2D scan) of the area around the indenter tip, and multiple 2D scans were performed at different loads. The objective of the experiment was to obtain a 2D map of the elastic stresses in the material below the indenter tip.

For the sake of this example, only one scan taken at a static load of *F* = 0.85 N is processed and discussed. This re-processing of published data was selected to give a baseline with which to compare results. This example was processed offline. The raw data consisted of a 2D scan of 21 by 23 datapoints (horizontal × vertical) arranged on a 500 ×500 nm grid. The data were acquired using a Photonic Science CCD detector with a 62 µm pixel size and at a photon energy of 14.73 keV (wavelength approximately 0.824 Å).

**Figure 3:**
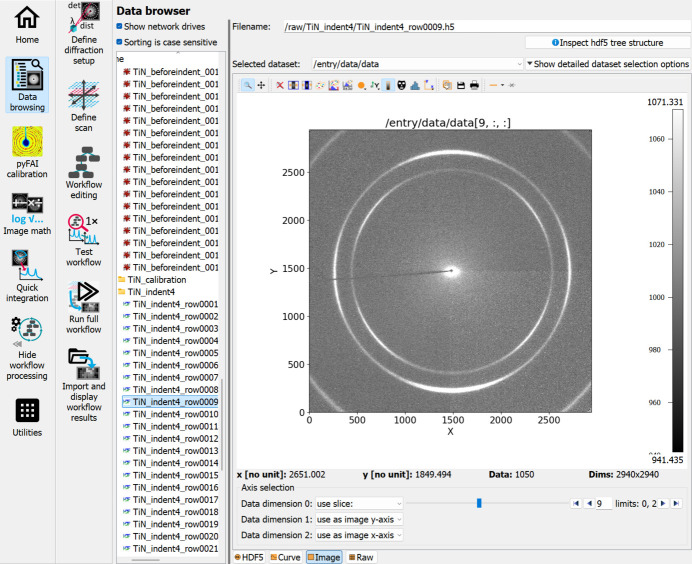
An exemplary image of the raw data used for this use case example, as displayed in the *pydidas* GUI. The innermost ring is the TiN 111 reflection and the second ring is the TiN 200 reflection, both of which can be used in the analysis.

A typical detector image shows two complete rings from the TiN 111 and TiN 200 reflections and partial rings from the TiN 220 reflection in the corners. A raw image is given in Fig. 3 ▸.

### 5.1. Calibration

To determine the exact geometry of the experiment (sample-to-detector distance, detector tilt, beamcenter) a detector calibration measurement was performed by scanning over a microcrystalline LaB_6_ sample to acquire a sufficient number of diffraction spots. Prior to the calibration process, the acquired calibration images were averaged using the *pydidas* image series operations tool to get a single file with all reflections.

The calibration was performed using the *pyFAI-calib2* application. The *calib2* application has been embedded in the *pydidas* GUI for convenient access. This leads to some minor visual changes in *pyFAI-calib2* but also allows an integrated use of the calibration result in *pydidas*. The calibration results can be copied into the experiment definition with a single button click. Fig. S2 shows exemplary screenshots of the calibration window embedded in *pydidas* and the standalone *pyFAI-calib2*. The use of *pyFAI-calib2* is described very well in the online help (https://www.silx.org/doc/pyFAI/latest/usage/cookbook/calib-gui/index.html#cookbook-calibration-gui) and will not be discussed further here.

### 5.2. Experimental setup and scan definition

The experimental setup frame, labeled ‘Define diffraction setup’ in the toolbar, is shown in Fig. S3. It features import and export buttons at the top and bottom, respectively. Also, a button allows parameters to be copied from the calibration. Parameters have been grouped into three categories: ‘Beamline X-ray energy’, ‘X-ray detector’ and ‘Detector geometry’. Because *pydidas* uses *pyFAI* for integrations, it uses the PONI geometry as introduced in *pyFAI*. A converter for changing *FIT2D*-style geometries to PONI is included to support working with previously calculated calibrations.

The beamline energy can be given either as photon wavelength or energy, and both values are displayed and updated upon changes to one of the two.

The full definition of the X-ray detector is required because *pyFAI*’s PONI geometry is given in absolute positions in SI units. Conveniently, *pyFAI* includes a number of stored mask layouts for the most widely used detectors with module gaps. Updating the detector mask allows additional hot or dead pixels to be masked. In addition to allowing direct editing of the detector geometry definition, the GUI includes tools to define the beamcenter position manually in a simplified geometry (without detector tilts) or to import a *FIT2D* geometry. Because the PONI position in metres from the detector corner is not directly intuitive, the derived beamcenter position in pixel coordinates is also given in the *pydidas* GUI.

The scan definition window is shown in Fig. S4. For the users’ convenience, it also features an explanation of the ordering for the scan dimensions. The global scan parameters are shown on the left and include the definition of scan dimensionality, scan base directory and scan naming pattern. Defining the data directory in the scan definition allows all workflow plugins to reference it without explicitly including paths in the workflow, which makes reusing workflows much easier. The scan naming pattern uses hashes (‘#’) as placeholders to mark counting variables. This nomenclature allows the use of fixed numbers, *e.g.* a sample reference number, in combination with counters. Each input plugin determines how it uses the naming pattern to create the full filename. Depending on the selected scan dimensionality, only the necessary parameter inputs for the selected dimensions are shown on the right. The GUI also includes small buttons to conveniently reorder the definitions of the scan dimensions.

### 5.3. Workflow editing

**Figure 4:**
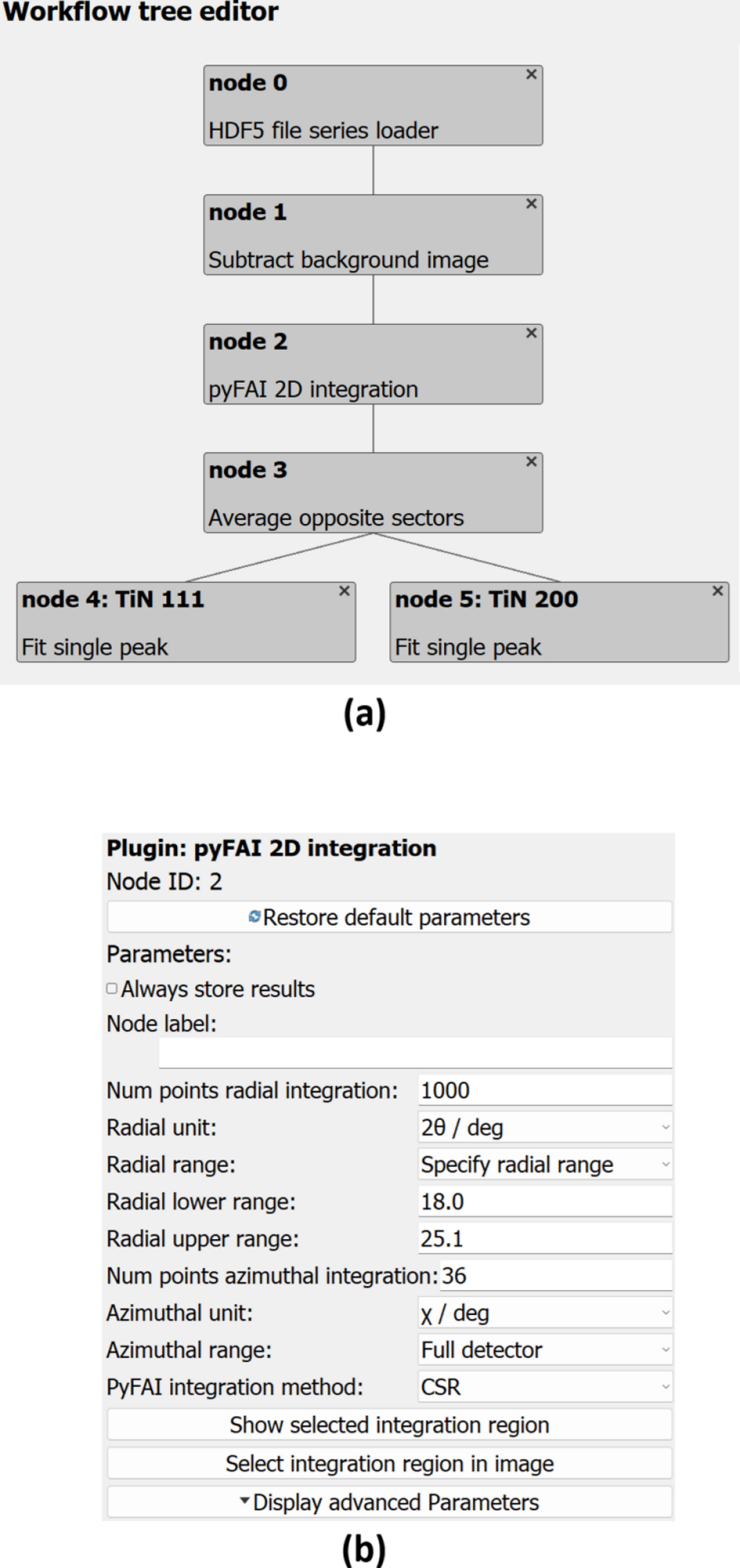
(*a*) Visualization of the workflow in the *pydidas* GUI. The individual nodes and their relationship show how data will be processed. (*b*) Exemplary configuration for a single plugin: the configuration is managed separately for each plugin to not overwhelm users with too many options. In this example, the configuration for the *pyFAI* 2D integration is displayed. Parameters for expert users are hidden to begin with but can be edited after using the ‘Display advanced Parameters’ button.

A view of the workflow editing frame is shown in Fig. S5. It has three main elements: a graphical visualization of the current workflow tree and its plugins as well as the plugin connections, a widget to browse all available plugins, and the plugin configuration on the right. Plugins can be reordered in the graphical visualization with drag and drop or through context menus. A single click on a plugin will show the corresponding plugin configuration on the right of the frame and highlight the selected plugin on the canvas. Fig. 4 ▸(*a*) shows the workflow used in this example.

Managing the parameter configuration for each plugin individually allows each plugin to have small numbers of parameters and clear association to the respective plugin. While most plugins have generic configuration widgets, this arrangement also allows unique configuration widgets to be implemented for specific plugins. For example, the output of peak fitting can be selected using checkboxes to allow all possible parameter combinations (peak position, width, height *etc*.). An example of the parameter configuration is given in Fig. 4 ▸(*b*).

The plugin browser at the bottom of the frame allows users to inspect the full list of available plugins, apply filters and display additional information for each plugin. All the information is taken automatically from the plugins’ docstrings and meta information. Custom plugins will be displayed automatically in the list if the plugins’ path is known to *pydidas*.

In the nano-indentation example, the workflow consists of an HDF5 file loader plugin, a plugin to subtract a background image to correct for the detector dark current, the azimuthal integration using *pyFAI*, and two fitting plugins for the TiN 111 and TiN 200 peaks.

### 5.4. Workflow testing

Before running the full workflow on all scan points, it is often reasonable to test the workflow on single input frames. The ‘Test workflow’ frame allows users to select single scan points and run the full processing workflow on these points. Points can be specified by either the global frame index, the detector image number or the scan position. Users can then inspect all intermediate results of all plugins. Fig. S6 shows a screenshot of the ‘Test workflow’ frame. All plugins in the workflow tree can be selected to inspect their intermediate results.

While intermediate results help to understand the data flow in the workflow tree, they do not allow the inner working of individual plugins to be inspected. If plugins specify that they give additional detailed intermediate results, these are also available here through a button. Fig. S7 shows a screenshot of the detailed results for a fitting plugin. The detailed results give an option to inspect intermediate runtime results for selected data points without keeping them available globally for all scan points.

In addition to visualizing intermediate results, the test workflow also allows plugin parameters to be tweaked on the fly and changes in the plugin’s output seen directly. Updated results are also propagated to all downstream plugins so that all results are consistent and up to date.

### 5.5. Running the full workflow

The ‘Run full workflow’ frame allows the workflow to be run for all scan points in independent background processes. Results are stored in the main process and can be displayed as they are produced. Fig. S8 shows a screenshot of the frame. Any results from the workflow can be displayed and the desired plugin is selected with a drop-down menu. This will display a textbox with additional information about the results, including data and axis labels and axis ranges. Controls below the textbox allow users to specify how data should be displayed (*i.e.* image or plots) and to select the display dimensions and slices. Slices can be selected both in axis indices and with data values. *Pydidas* will find the closest index to the selected data value to display.

**Figure 5:**
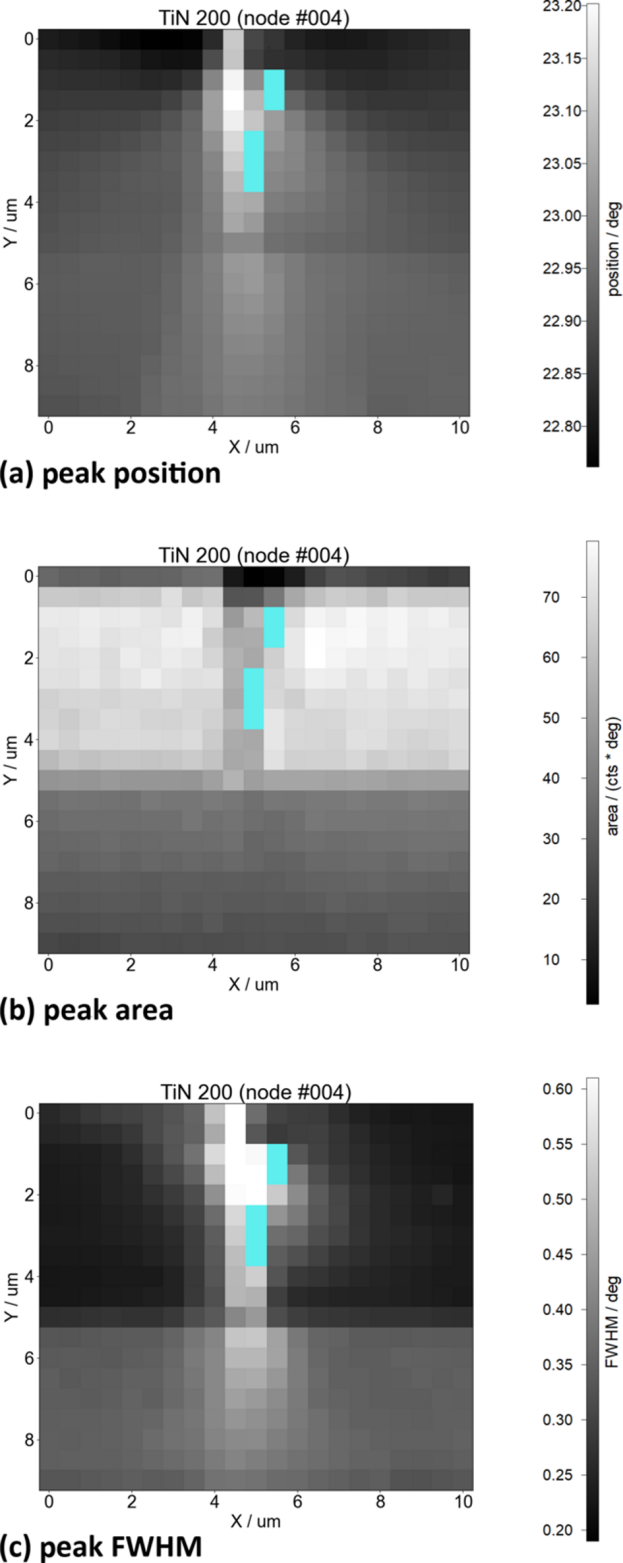
Processing results of the TiN 200 reflection peak fit from the use case example: (*a*) peak position; (*b*) peak area; (*c*) peak FWHM. The positions where the fit quality did not achieve the required threshold are marked in cyan for easy identification.

During processing, the data display will automatically be updated as results come in from the worker processes. Data can be exported in HDF5 format for later use in *pydidas* or other applications. In this example, the data in Fig. 5 ▸ show the results at the azimuthal χ = 90° position, as shown in *pydidas*. The results clearly show the layered deposition of the TiN coating and how the indenter has left its mark on the sample. For the scientific discussion of the sample, readers are referred to the work of Zeilinger *et al.* (2016 ▸).

**Figure 6:**
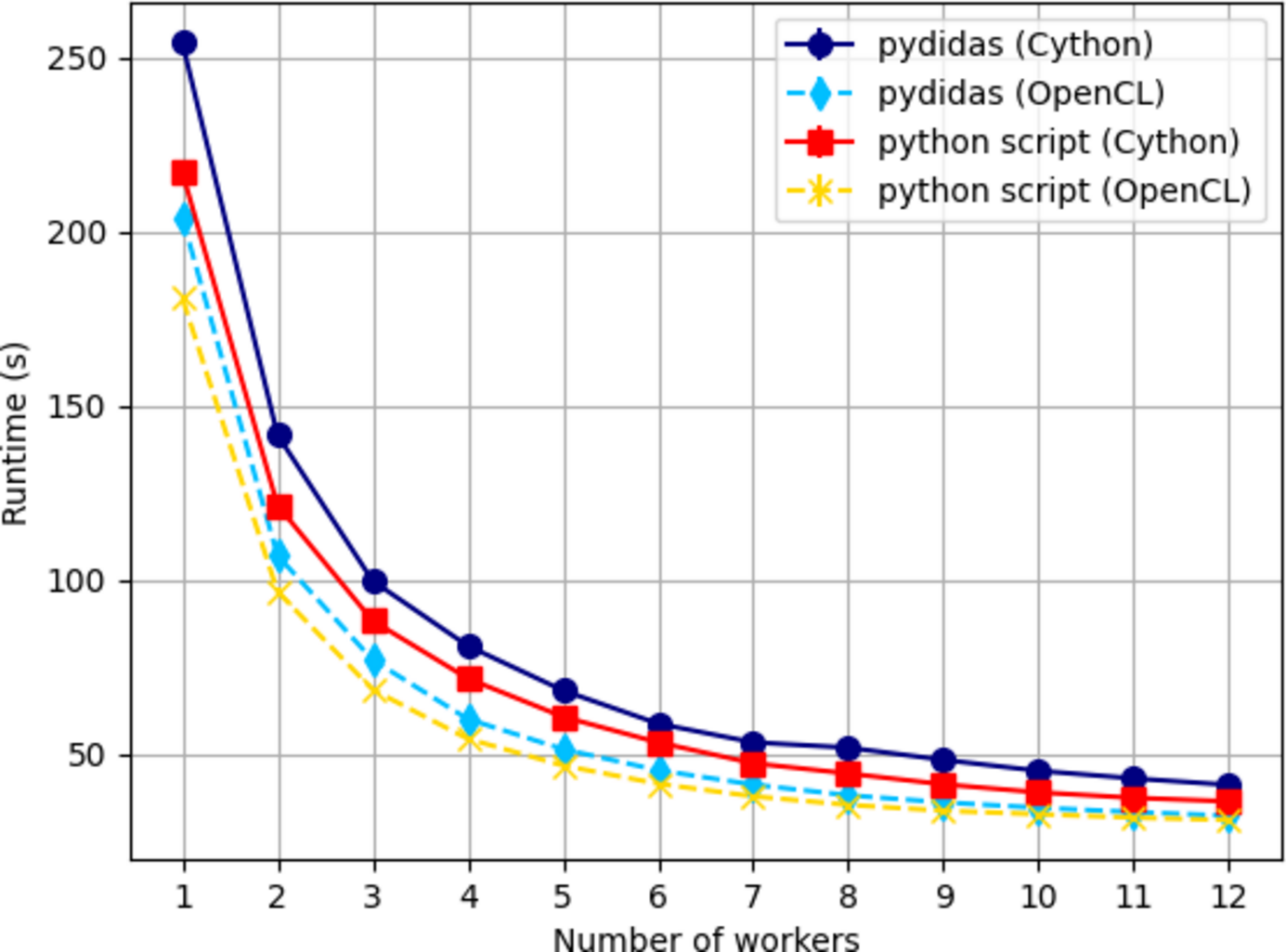
Runtime test for processing of all 399 data points in the scan. Compared with a direct Python implementation (using *pyFAI* and *scipy*), *pydidas* introduces an overhead of approximately 10% which includes data transport between the processes.

The processing has been benchmarked in comparison with a direct processing in Python on a local workstation. Details about the comparison and machine are given in Appendix *C*[App appc]. A graphical representation of the results is shown in Fig. 6 ▸. The processing speed in *pydidas* is comparable to direct calling of *pyFAI* and *scipy* least-squares peak fits with an overhead of approximately 10% of the processing time. This includes, however, the data transport between the processes and gathering and writing of the results in an HDF5 file. In the script implementation, only the raw processing has been analyzed.

## 6. Summary

*Pydidas* has been developed as a user-friendly integrated toolbox for the automated analysis of diffraction data. It allows beamline users to perform fast analysis on site during experiments and to continue analysis in the same tool later at their home institutions. The open architecture allows inclusion of specialized loaders for additional file and path structures, and additional processing plugins for functionality which is not included in the *pydidas* core functionality.

Special emphasis has been placed on an intuitive user interface to extend the potential user community for XRD experiments by offering improved analysis software.

We are actively promoting *pydidas* to our users at beamlines P03, P07 and P07B, and the majority of user groups at these beamlines actively use *pydidas* for their analysis. The feedback from our users has been overwhelmingly positive and they have reported a significant reduction in the working time they need to reduce and analyze their experimental data.

At our beamlines, integrating *pydidas* in the alignment of advanced experiments, for example for conical slits, has reduced the setup time significantly, which improves the beamline efficiency and allows beamline scientists to spend more time with the user groups.

In addition, we are in discussion with other beamlines to determine their specific requirements for starting to use *pydidas*.

## Supplementary Material

Supplementary figures. DOI: 10.1107/S160057672500398X/yr5156sup1.pdf

Pydidas source code: https://doi.org/10.5281/zenodo.13788623

## Figures and Tables

**Table 1:** Separation of the configuration into three different groups

Category	Content
Experiment	Beamline setup:
	X-ray energy/wavelength
	X-ray detector configuration (detector model)
	Detector mask file, if applicable
	Detector geometry

Scan	No. of scan dimensions
	No. of points in each dimension
	Scan base directory
	Scan naming pattern
	Metadata:
	Scan title
	Scan axis names, units and ranges

Workflow	Processing plugins used
	Plugin data flow
	Plugin configuration
	Export of results

**Table 2:** Overview of the available standard frames in *pydidas*

Frame title	Content
Data browsing	Explore files via a file system browser and display the file contents as 1D graphs or 2D images via a visualization widget
*pyFAI* calibration	Calibrate the experimental detector geometry via an integrated version of the *pyFAI-calib2* tool
Image math	Perform arithmetic operations on single images or pairs of images and apply operators (*e.g.* thresholding, log) on images
Quick integration	Perform an interactive beamcenter selection in an image and a quick integration with a simplified geometry (no tilts)
Utilities	Access various utilities like averaging images, maskediting, configuration

Workflow processing	
Define diffraction setup	Configure the experimental parameters, as detailed in Table 1 ▸
Define scan	Configure the scan, as detailed in Table 1 ▸
Workflow editing	Define and edit the workflow, as detailed in Table 1 ▸
Test workflow	Test the workflow on a single scan point and inspect all intermediate results
Run full workflow	Run the full workflow for all scan points and visualize results
Import and display of workflow results	Import workflow results from previous runs and display the resulting data

## Data Availability

The source code for *pydidas* is available under the GPL3 open-source license via https://github.com/hereon-GEMS/pydidas and https://doi.org/10.5281/zenodo.13788623 with online documentation at https://hereon-gems.github.io/pydidas/.

## Appendix A Plugin example

A minimal example for a custom plugin is given below. Plugins need to inherit from one of the base classes InputPlugin, ProcPlugin (short for processing plugin) or OutputPlugin. As a bare minimum, the plugin class needs to define a plugin_name and default_params, if required. The pre_execute and execute methods are required as well. pre_execute performs static tasks that need to be run once before running batch processing and the execute method will be called for each processing point. It receives the input data and any kwargs from the earlier plugins and needs to return a single dataset and dictionary again. More detailed information on customization is available at https://hereon-gems.github.io/pydidas/dev_guide/dev_guide_plugins.html.[Chem scheme1]

Custom plugins will be automatically identified and loaded by *pydidas*. *Pydidas* will search for plugins in user plugin paths and import all plugins that are child classes of the base plugin classes. An example for setting a custom path is given below:[Chem scheme2]

Note that multiple paths can be specified and they need to be separated by a double semicolon. In the GUI, the user plugin path is included in the user options. Plugins can then be accessed as in-built plugins:[Chem scheme3]

## Appendix B Command-line interface example

The configuration for all context objects is handled through an identical interface. Parameters can be accessed by their reference keys. The following example shows how to configure the experiment in the command-line interface:[Chem scheme4]

## Appendix C Runtime benchmarks

The runtime tests were performed on a workstation with an Intel Xeon W7-3465X with 28 physical CPU cores and 256 GB RAM and a single Nvidia RTX A5000, running Windows 11 (version 23H2).

The processing pipeline included the following: (i) loading single frames from HDF5 files; (ii) subtracting a detector background image with thresholding at 0; (iii) running *pyFAI* integrate2d with 1000 × 36 points in the radial range of  = [18°, 25°], and using the ‘bbbox, csr, opencl/cython’ engine. The azimuthal intervals [18.5°, 21°] and [21°, 23.5°] were used to fit a Voigt profile (scipy.special.voigt_profile) including a constant background to the data. These two fits have been performed for each of the 36 radial bins.

In the direct script implementation, a worker pool (multiprocessing.Pool) of *n* workers was used and the Pool.apply_async method was used to process each frame. The calling argument was the frame number to keep data transfer between the processes small. Each worker then determined the respective frame to process on the basis of the frame number and ran the full processing pipeline.
