# Supplementary material for: Pydidas: a tool for automated X-ray diffraction data analysis
Source: J Appl Crystallogr. 2025 Jun 16;58(Pt 4):1476–85. doi: 10.1107/S160057672500398X (PMC12321008; doi:10.1107/S160057672500398X)
Supplement: Supplementary file 1 [file j-58-01476-sup1.pdf]

JOURNAL OF APPLIED CRYSTALLOGRAPHY

Volume 58 (2025)

Supporting information for article:

Pydidas: a tool for automated X-ray diffraction data analysis

Malte Storm, Peter Staron and Christina Krykwa

Pydidas: A tool for automated X-ray diffraction data analysis — Supplementary Figures

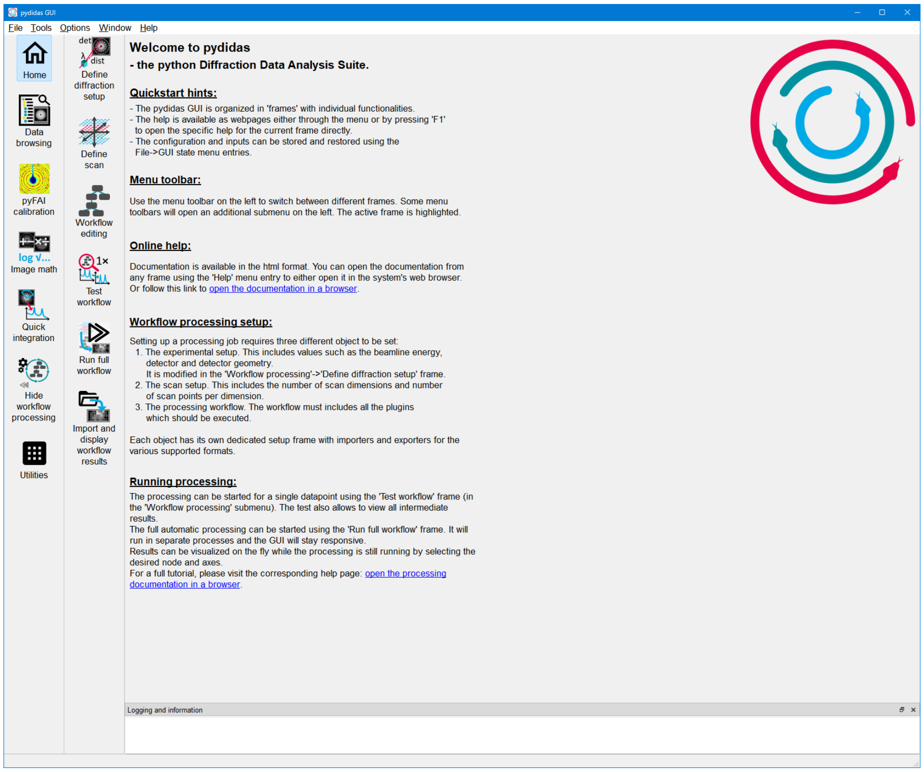

Figure S1: The pydidas main window after start-up. The navigation is handled through the toolbar with labeled buttons on the left and the canvas on the right is used for displaying the selected content

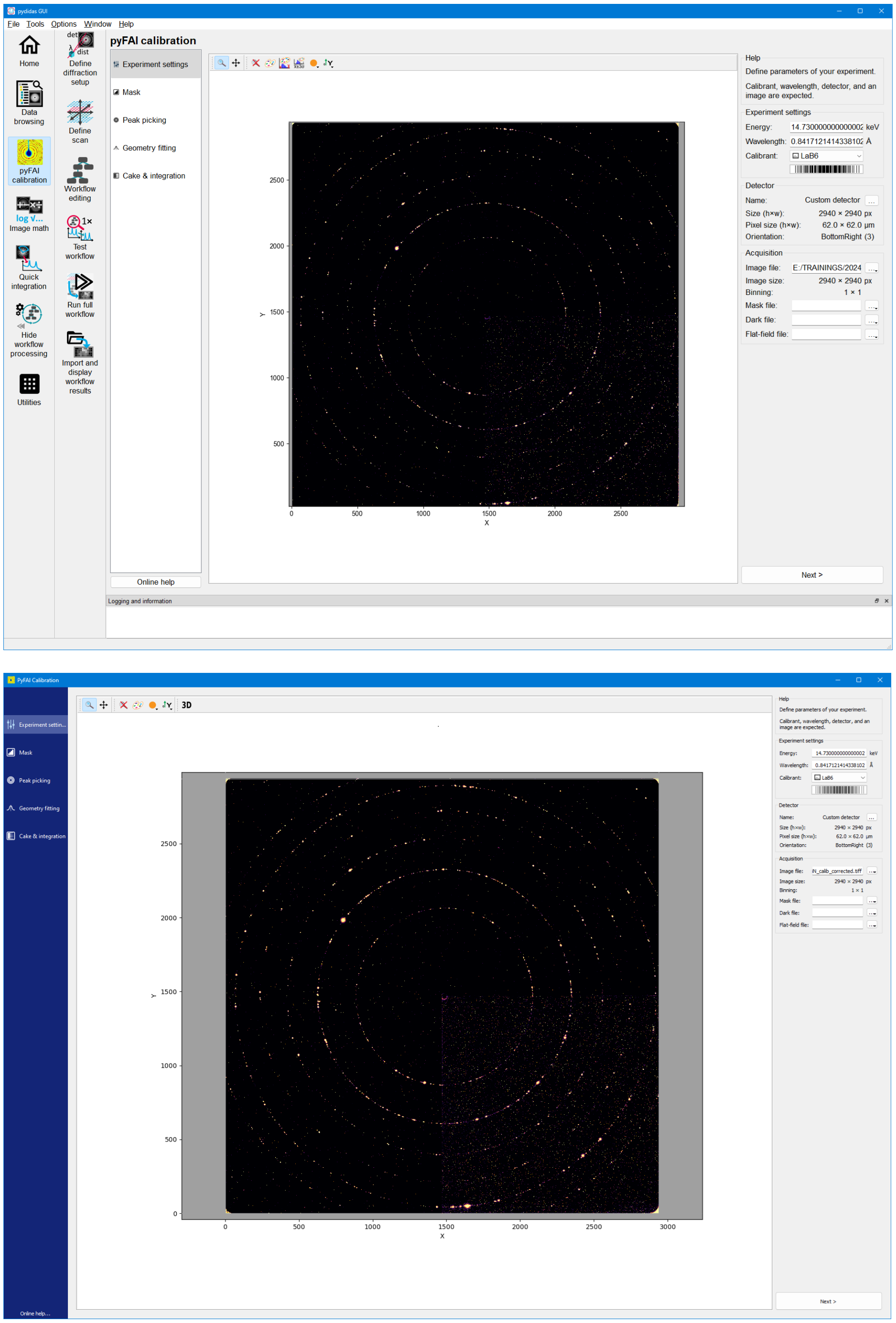

Figure S2: Side-by-side views of the geometry fitting task of the pyFAI-calib2 in pydidas (top) and as a stand-alone application (bottom).

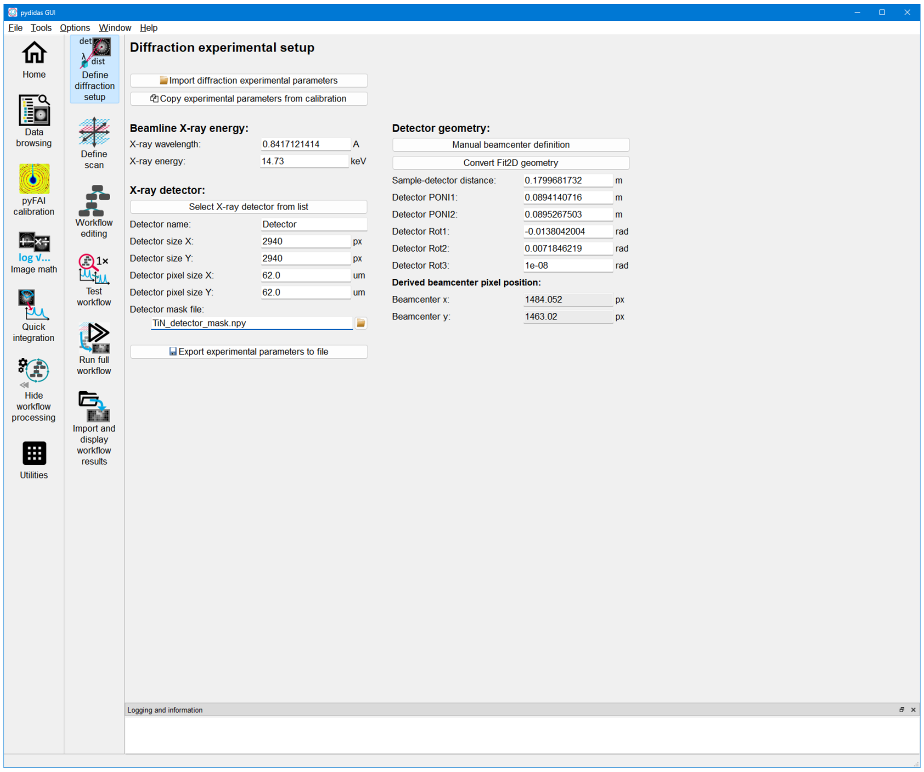

Figure S3: Screenshot of the diffraction experimental setup. The frame includes import of saved definitions at the top and export buttons at the bottom of the frame and allows the configuration of its parameters in the central region. Buttons are used to access additional functionality like defining the X-ray detector through its model (using pyFAI functionality), manually setting the beamcenter or importing a geometry by its Fit2D-specification.

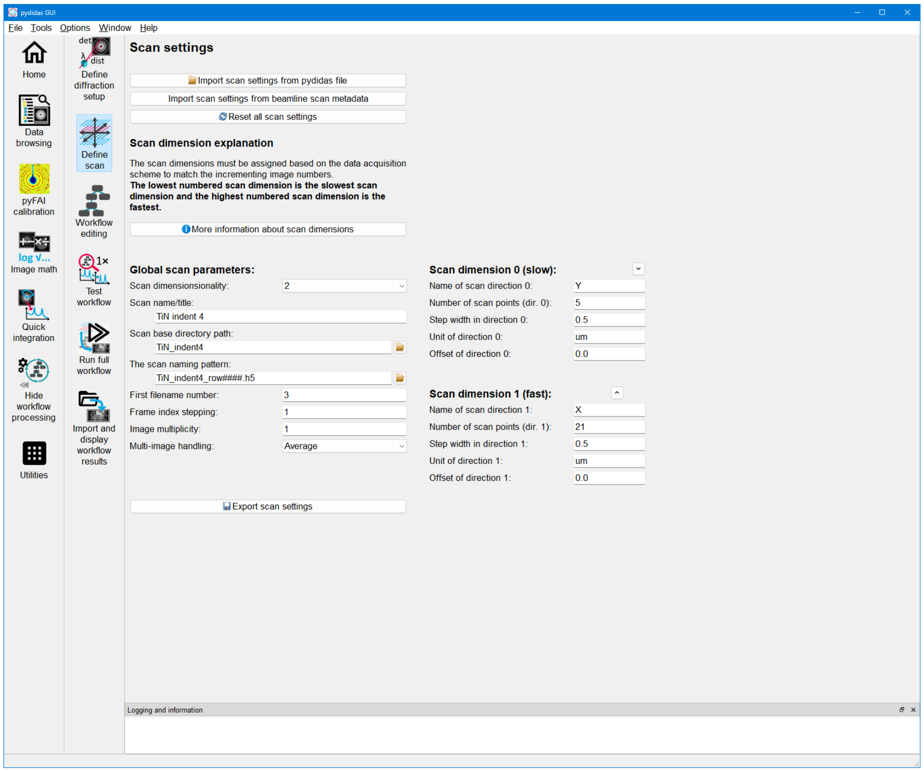

Figure S4: Screenshots of the scan setup. The frame includes import of saved definitions at the top and export buttons at the bottom of the frame and the configuration of its parameters in the central region. An explanation about the ordering of scan dimensions is included for quick reference by the user. Scan dimensions can also be moved up and down by the small respective buttons in the top right corner of the definition for each dimension.

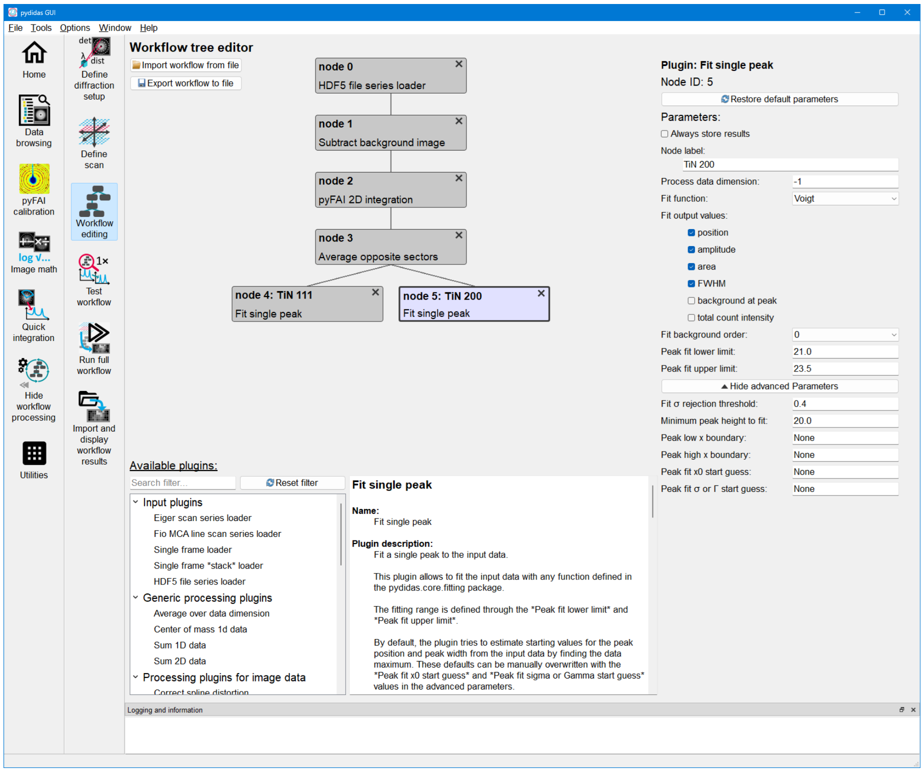

Figure S5: Screenshot of the workflow editing frame. The top left canvas holds the visual representation of the current workflow tree, the bottom left widget shows the list of available plugins and further information about the plugins. The right area is taken up by the configuration of the actively selected plugin which is also marked with a colored background and bold frame on the top right

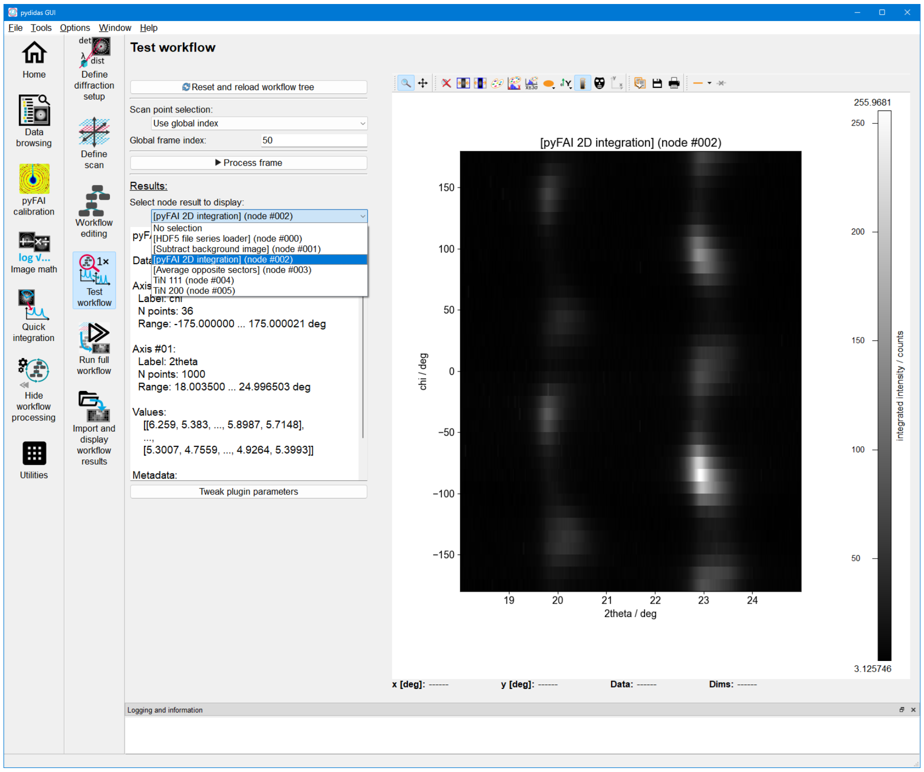

Figure S6: Screenshot of the Test workflow frame. The scan point can be selected on the top left (in the screenshot through the global frame index). The different plugins can be selected in a drop-down menu (opened in the screenshot) and detailed information about the results is shown in a text box on the left as well as in a plot on the right.

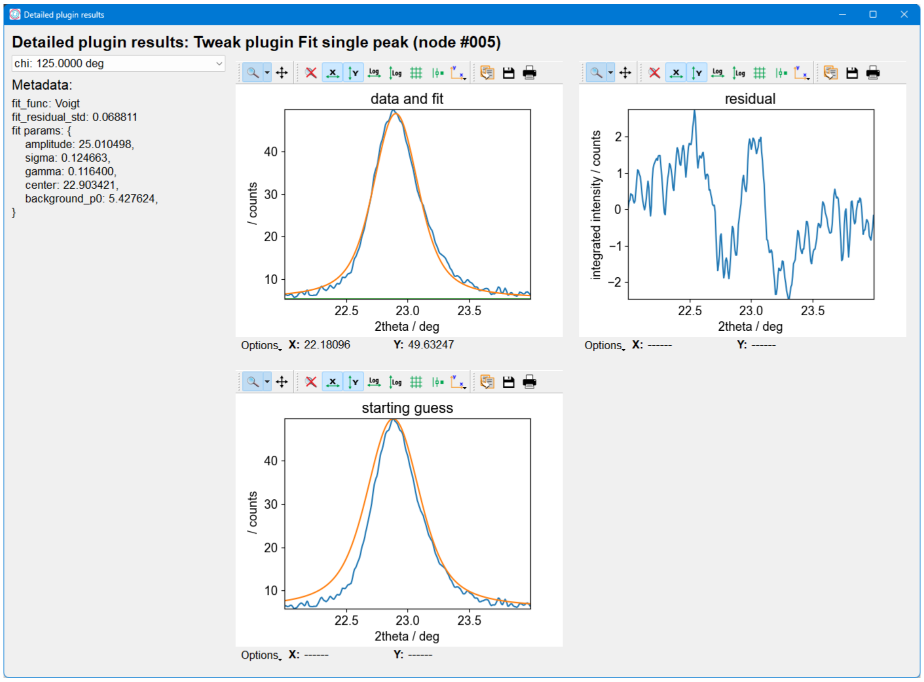

Figure S7: Screenshot of the detailed results for a fitting plugin. The panel on the left allows to select the azimuthal chi position to inspect and the text shows the fitted parameters. The three plots show the data and resulting fit (top left), the residual (top right), and the starting guess for the fit (bottom left).

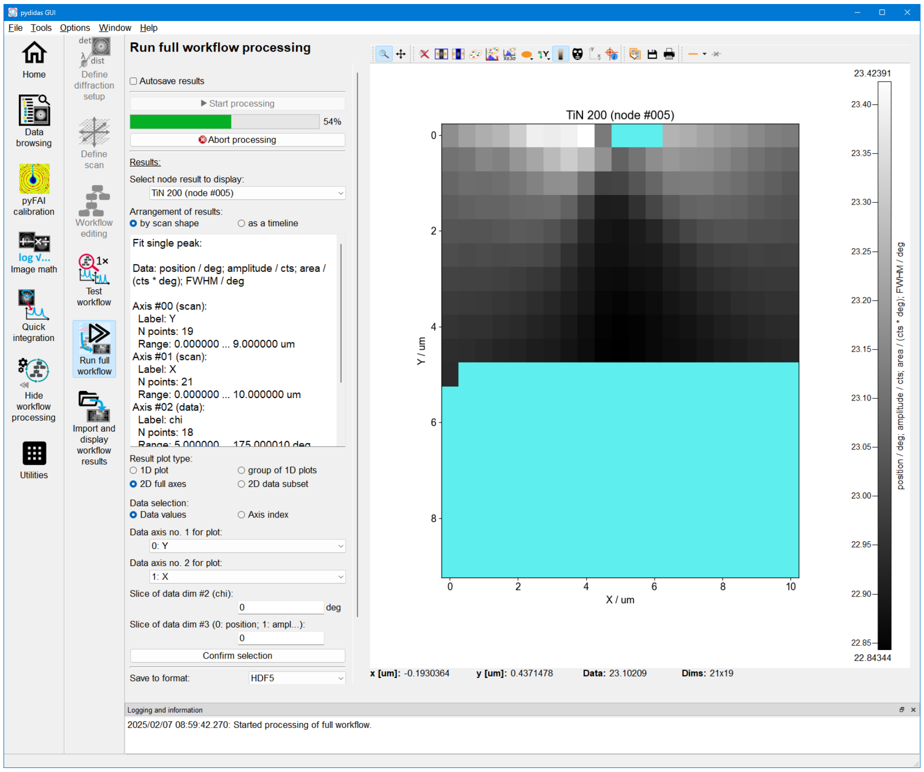

Figure S8: Screenshot of the run full workflow frame. The left part of the frame includes the processing controls at the top, data selection below it and the export dialog at the bottom (not visible). The right part of the frame shows an image of the selected dataset. The cyan-colored parts show invalid data. The points at the top indicate that the fit did not meet the defined quality and the part at the bottom of the image is missing data because the processing is not yet finished.
